# The Added Value of Endoscopic Micro-Inspection in Microvascular Decompression for Trigeminal Neuralgia and Hemifacial Spasm: Literature Review and Single-Center Experience

**DOI:** 10.3390/neurolint18040066

**Published:** 2026-03-31

**Authors:** Alexandra Mihaela Pătrășcan, Felix Mircea Brehar, Radu Mircea Gorgan, Viorel Mihai Prună

**Affiliations:** 1 Department of Neurosurgery, “Carol Davila” University of Medicine and Pharmacy, 050474 Bucharest, Romania; alexandra-mihaela.patrascan@drd.umfcd.ro (A.M.P.); radu.gorgan@umfcd.ro (R.M.G.); viorel.pruna@umfcd.ro (V.M.P.); 2 Department of Neurosurgery, “Bagdasar-Arseni” Clinical Emergency Hospital, 041915 Bucharest, Romania

**Keywords:** microvascular decompression, neurovascular compression syndrome, trigeminal neuralgia, hemifacial spasm, QEVO, endoscopy

## Abstract

**Background:** In the last few decades, microvascular decompression has been proven to be one of the best therapeutic options in the management of neurovascular compression syndromes, especially trigeminal neuralgia, hemifacial spasm, and glossopharyngeal neuralgia. However, higher rates of recurrences and morbidities have been recorded postoperatively. In the thorough search for better solutions, the option of adjuvant QEVO^®^ endoscopy has arisen as a very promising alternative. **Methods:** In this study, a retrospective single-center observational analysis was conducted, comprising patients who underwent microvascular decompression for trigeminal neuralgia, hemifacial spasm, and glossopharyngeal neuralgia at our institution, between January 2020 and November 2025. Demographical data and outcomes of therapeutic management were statistically analyzed and presented accordingly. **Results:** A total of 40 patients diagnosed with neurovascular compression syndromes were neurosurgically treated in our center, and the most common diagnosis was represented by trigeminal neuralgia, identified in 32 patients (80%). Another five (12.5%) patients underwent microvascular decompression for hemifacial spasm, two (5%) patients were treated for combined trigeminal neuralgia and hemifacial spasm, and one patient (2.5%) for glossopharyngeal neuralgia. Arterial conflict was the triggering factor in the majority of cases, and no postoperative mortality was recorded. In patients treated using adjuvant QEVO endoscopy, the identification of hidden conflicts may be facilitated. Furthermore, the use of the QEVO endoscope allowed the identification of additional neurovascular conflicts and influenced intraoperative management in a subset of patients. **Conclusions:** Notwithstanding the medical literature suggesting that the main influential factor for therapeutic success is the vessel type and the pattern of compression, many authors identified the cornerstone of favorable outcomes as being endoscopic assistance. Nevertheless, this adjuvant factor has had a positive impact on the majority of patients.

## 1. General Overview of Neurovascular Compression Syndromes

Neurovascular compression syndrome (NVCS) is a condition characterized by the direct contact of a vessel with the cisternal portion of a cranial nerve, causing mechanical irritation [1]. Although the vast majority of patients exhibit significant disabling symptoms triggered even by simple daily activities, asymptomatic patients were also reported as being diagnosed incidentally after neuroimaging investigations [2]. The most commonly reported forms of NVCS are trigeminal neuralgia (TN), hemifacial spasm (HFS), glossopharyngeal neuralgia (GPN), and vestibular paroxysmia (VP) [3]. Other forms, with very rare accounts reported in the medical literature, include oculomotor nerve palsy [4], superior oblique myokymia due to NVCS of the trochlear nerve [5,6,7], abducens nerve palsy [8], and vagoglossopharyngeal neuralgia [9]. Table 1 summarizes the epidemiology of the most common NVCSs reported to date. It is worth mentioning that higher variations can be reported due to data collection in different countries and with different criteria [10].

Concerning the diagnosis of NVCS, it is important to mention that the advancement and availability of neuroimaging methods nowadays have led to a better diagnosis of this condition, even in asymptomatic individuals [18]. Nevertheless, in many cases, the diagnosis remains primarily clinical, while the neuroimaging examinations mainly exclude secondary causes, assessing and characterizing the neurovascular conflict [18].

It has been demonstrated that advanced MRI sequences, such as 3D CISS, 3D FLASH, or 3D FIESTA, are the best option for visualizing NVCS, and imaging at 3.0 Tesla is superior in detecting small vascular culprits [19]. While diffusion tensor imaging has shown promising results as it identifies microchanges in the structure of the trigeminal nerve [19], 3D CISS MRI with multiplanar reconstruction has been proven to be superior to MR angiography in the identification of patients with NVCS [20]. However, in cases of the unavailability or contraindications in these methods, the European Academy of Neurology guideline on trigeminal neuralgia recommends the use of trigeminal reflexes, especially to distinguish secondary TN from primary TN, and strongly recommends against the use of evoked potentials to diagnose a secondary TN [18].

Regarding the therapeutic management in NVCS, the available options may differ based on the type; thus, we will discuss them separately.

**Figure 1:**
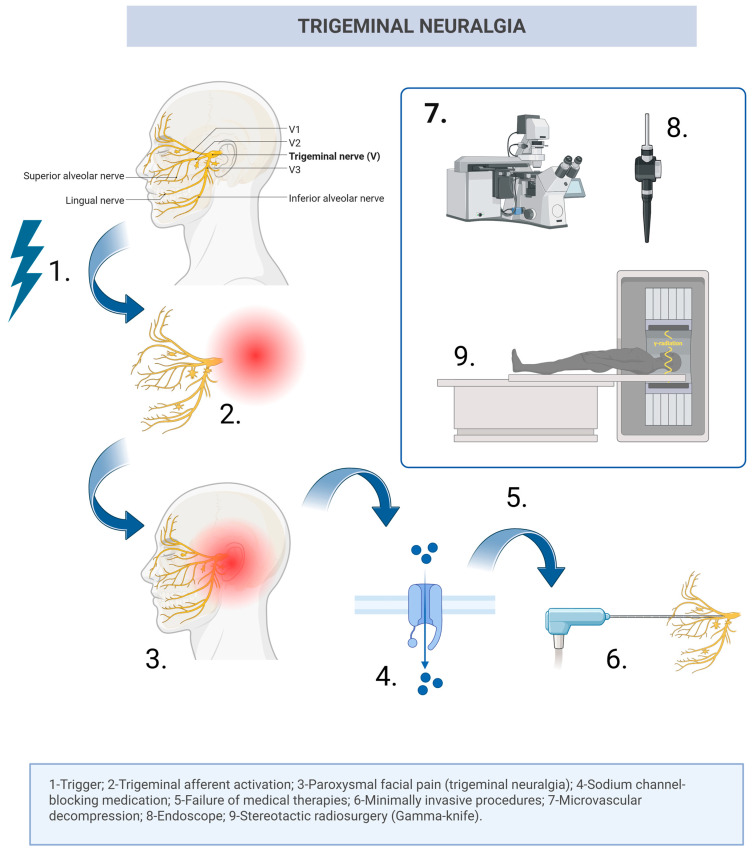
Schematic illustration of the pathophysiological and therapeutic sequence in trigeminal neuralgia. Created with BioRender. Steps 6 and 9 are not always necessary and are of choice, as many patients choose MVD surgery directly, given its excellent results (Created in BioRender. Mihaela Patrascan, A. (2026) https://BioRender.com/q1kpt12).

In TN, the first-line treatment is represented by sodium channel blockers, and for long-term use, carbamazepine and oxcarbazepine remain the most effective, especially in the early stages of the condition [18]. Nevertheless, despite its effectiveness, oral medication sometimes fails to keep the patients asymptomatic or can lead to insufferable side effects. In this case, TN is considered refractory to first-line therapy [21] and minimally invasive procedures, such as partial sensory rhizotomy and internal neurolysis, gamma-knife radiosurgery (GKR), radiofrequency thermocoagulation, balloon compression, glycerol rhizolysis [18,22], or microvascular decompression, should be considered. While some authors may recommend the use of neuroablative options in patients with no detected conflict on neuroimaging examinations [21], various studies have proven the superiority of MVD in comparison with any other therapeutic option, reaching the highest percentage of patients with long-term good results among all of the options [23,24,25,26,27,28]. While no significant differences were obtained when comparing Teflon versus Ivalon in MVD [29], future directions point out Teflon-free MVD techniques; however, further studies are needed in order to validate the results [30]. Figure 1 summarizes the pathophysiological and therapeutic sequence in patients with TN.

When it comes to HFS, the patients present with a unilateral facial muscle spasm, which negatively impacts their quality of life, specifically given the factor of social embarrassment [31]. Although the treatment of this condition often takes the form of botulinum toxin injection, with good short-term results [32], superior results were obtained after MVD surgery [33]. MVD in HFS addresses the root cause of the condition and allows the neurosurgeon to separate the offending vessels from the facial nerve and the root exit zone [34], with an efficacy rate of up to 90% [35]. Nevertheless, it is important to mention that the reported complications remain meaningful, highlighting the importance of neurosurgical experience and the need for adjuvant visualization tools in order to obtain the best outcome possible [35].

Concerning GPN, patients often present with severe pain, located unilaterally in the throat, tonsillar fossa, tongue, or ear, sometimes triggered by simple daily activities [36,37]. Similar to TN, the therapeutic options include oral medications, but in most cases, this has been proven insufficient [37], leading to MVD surgery if the patient is considered a good candidate [38]. In a recent study from 2025 by Hajikarimloo et al., the authors demonstrated the efficacy of MVD surgery in GPN, reporting minimal perioperative morbidity and long-term results [39]. Table 2 summarizes the reported outcomes in the medical literature for patients treated using MVD for GPN.

Vestibular paroxysmia is an NVCS represented by short attacks of vertigo and dizziness, sometimes with auditory problems, which may last up to a few minutes [16]. Similarly to other types of NVCS, oral medications have been proven to be effective, with oxcarbazepine being reported as having good long-term results [45].

Regarding the other less common types of reported NVCS, the medical literature on the matter is scarce; thus, further clinical studies are encouraged and expected in the future.

## 2. Recurrence, Reintervention, and Why Visualization and Endoscope-Assisted Micro-Inspection Matter in MVD

Notwithstanding its successful results and great overall long-term outcomes, MVD surgery has never been risk-free. Some patients present with recurrences after surgeries that seemed successful at first glance and in the short term, with incomplete decompression, prosthesis displacement, or postoperative tissue adhesions being the most frequently reported cases of recurrences [46]. While in patients with recurrences, a repeated MVD surgery can provide good long-term results, reintervention also comes with higher risks of perioperative complications, as reported by many authors in the medical literature [47,48,49].

To date, one of the best preemptive strategies to avoid recurrences following MVD surgery is adjuvant intraoperative endoscopy. This approach addresses the main limitation of incomplete visualization of the involved anatomical structures of neurovascular conflict, providing a better visualization “around the corner” [50]. Moreover, the endoscopic tool is precisely important in neurosurgical cases when there is a prominent suprameatal tubercle that impedes the visualization of Meckel’s cave using only an operative microscope, improving the detection of hidden neurovascular conflicts [51].

The main goal of endoscopy in MVD is to maintain the ergonomic advantages of bimanuality, while additionally providing a visualization “around the corner”, in order to inspect or confirm the nerve decompression [51]. As already presented in Table 3, some authors have concluded that endoscopic MVD can have similar pain relief and complication rates when compared to classical MVD; however, the real efficacy of the use of an endoscope is highlighted in cases of intricate anatomy in which sometimes even more conflicts are revealed [50,52]. Thus, the primary goal and benefit of incorporating an endoscope is better visualization, without leaving certain anatomical structures hidden [53]. When using an angulated endoscope in NVCS in the retrosigmoid area, the nerve can be seen in its entire cisternal segment, which includes the attachment to the brainstem and the side behind the nerve. In TN, for example, a 30- or 45-degree endoscope can visualize the nerve’s root from the porus trigeminus to the brainstem [51,54]. Table 3 summarizes the main articles in the medical literature that test and report the importance of endoscopy in NVCS.

## 3. Materials and Methods

This retrospective single-center study aims to assess the outcomes of 40 patients who presented with NVCS and were treated by endoscope-assisted MVD surgery (QEVO endoscope available at our institution, Carl Zeiss Meditec AG, Oberkochen, Germany) versus classical MVD. All of the patients were treated in the Neurosurgical Department of Clinical Emergency Hospital Bagdasar-Arseni, Bucharest, Romania, between January 2020 and November 2025, and written consent was obtained from all participants. The current study was approved by the Research Ethics Committee of “Bagdasar-Arseni” Clinical Emergency Hospital (protocol code: No 2674 and date of approval on 22 January 2026). Data were collected from the hospital’s databases and individual medical records. To select the patients, we searched the hospital’s digital databases for the terms “neurovascular compression syndrome”, “trigeminal neuralgia”, “hemifacial spasm”, and “glossopharyngeal neuralgia”. Subsequently, we evaluated each patient’s physical files and performed a thorough appraisal of their data, reviewing elements such as demographics, neuroimaging features, clinicopathological data, neurosurgical treatment, and outcomes.

The inclusion criteria were as follows: (1) patients over 18 years old with a confirmed diagnosis of NVCS; (2) patients who underwent MVD surgery at our institution, performed by neurosurgeons with comparable training and experience, regardless of whether the patients had previously undergone other treatment procedures; (3) availability of intraoperative data, including the use of the endoscope in a subset of cases. The exclusion criteria were as follows: (1) incomplete clinical, imaging, or operative data; (2) patients with secondary causes of NVCS.

Intraoperative findings were assessed based on operative reports and intraoperative video recordings, when available. Postoperative outcome data were obtained from clinical follow-up records and, when necessary, supplemented by patient-reported information collected during routine follow-up. A good outcome was defined as BNI grade 1 for TN and GPN, and complete postoperative resolution of HFS.

### Statistics and Replicability

Patients’ clinical and paraclinical features, clinical course, therapeutic strategies, and outcomes were extracted from medical records and further assessed. The collected data were coded/parsed and analyzed using Microsoft Excel version 16.89.1 (Microsoft Corporation, Redmond, WA, USA), Python 3.10.11 (Python Software Foundation, Wilmington, DE, USA), and GraphPad Prism version 10.2.1 (GraphPad Software, San Diego, CA, USA). Statistical significance was defined as a *p*-value < 0.05, while analyses involving rare outcomes and small subgroup sizes were interpreted as exploratory and hypothetical. All collected data were anonymized to protect patient confidentiality and ensure compliance with ethical research standards.

## 4. Results

### 4.1. Demographic Profile, Clinicopathological Characteristics, and Correlation Analysis

After careful evaluation, a total of 40 patients fulfilled the selected criteria and were included in the study. The demographic profile and cohort characteristics are presented in Table 4.

### 4.2. Statistical Analysis and Correlations

We analyzed patients with TN versus HFS before the neurosurgical intervention and at the moment of the intervention in order to determine whether there are important differences or similarities between the two groups. For the initial analysis, we included only pure TN and HFS cases, and we excluded GPN and the mixed cases, as shown in Table 5. This choice stems from the fact that, although TN and HFS are neurovascular compression syndromes treatable with the same intervention—microvascular decompression—they are anatomically and surgically different. Not only do they involve different cranial nerves and different anatomical corridors, but the root entry/exit zone and surrounding structures are also different. In microvascular decompression for trigeminal neuralgia, an enlarged suprameatal tubercle may sometimes limit the inspection of the trigeminal nerve, while in hemifacial spasm, the intervention may be difficult due to the presence of multiple vessels, some with complex anatomical configurations. These differences may influence surgical exposure, difficulty, and the risk of complications, and allow us to evaluate whether endoscopic assistance in microscopic microvascular decompression improves the visualization and identification of residual or hidden conflicts in both NVCS.

The *p*-values show if the recorded difference is statistically significant or just an occurrence. However, it is important to mention that there are only 5 patients with HFS; thus, the *p*-values could be unstable and must be cautiously interpreted. Table 5 summarizes all the recorded differences, and the rows highlighted in bold were statistically significant. The legend of each figure details the subset of patients analyzed.

**Figure 2:**
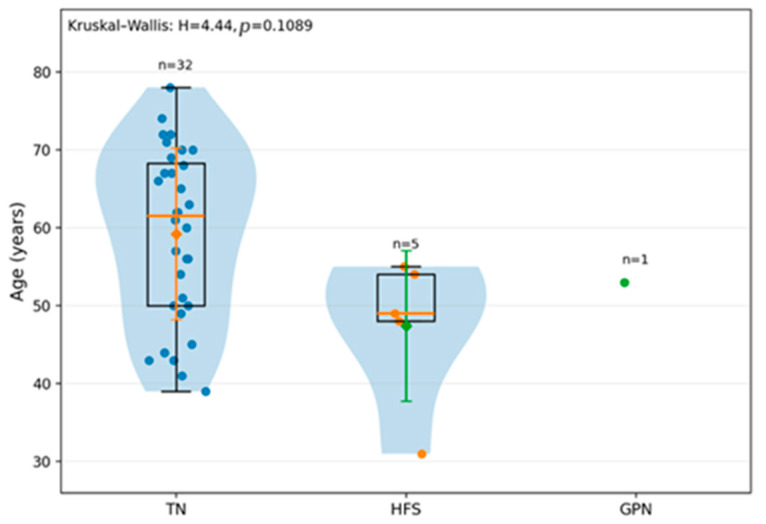
Age distribution by type of NVCS in our study group (TN versus HFS versus GPN) (*p* = 0.1089).

Although when it comes to age distribution in TN versus HFS, the difference was statistically significant (*p* = 0.043), when analyzing the age distribution in the entire group, including GPN, we found that patients with TN are generally older than patients with HFS (mean age 59 years versus 47 years), as shown in Figure 2. GPN cannot be interpreted as showing any distribution, given that there is only one patient. However, the conclusion does not reach statistical significance, probably because of the small groups of patients with HFS and GPN.

**Figure 3:**
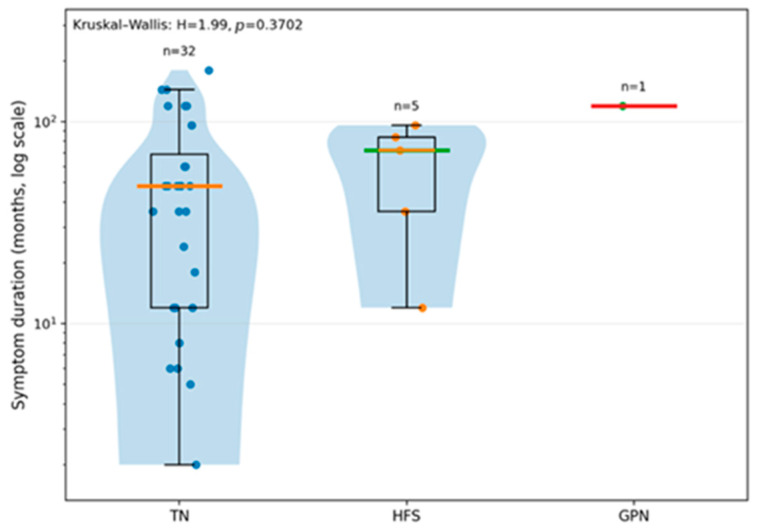
Symptoms duration by type of NVCS (TN versus HFS versus GPN) (*p* = 0.3702).

Regarding the symptom duration in our study population, it has been concluded that patients with HFS tend to have symptoms that last longer than those in patients with TN (Figure 3). The y-axis logarithmic scale allows the variables to be more visible, given that the symptom duration is usually very asymmetrical. However, the overall comparison yields a result that is not statistically significant (*p* = 0.3702), most likely due to the small number of patients with HFS and GPN.

**Figure 4:**
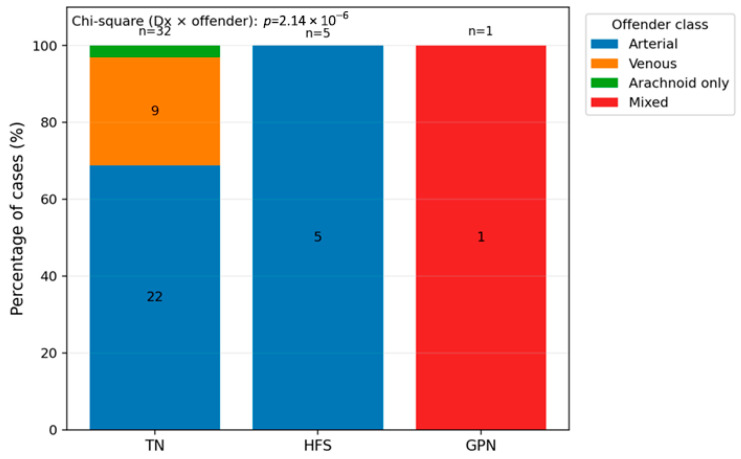
Intraoperative offender type by diagnosis (type of NVCS) in our study population (*p* < 0.0001).

The offender vessels were also analyzed in our cohort, and we found that an arterial offender was the most common (Figure 4). In patients with TN, arterial offender was recorded in 68.8% of cases, venous in 28.1% of the cases, and arachnoid adhesions were present in one patient (3.1%). In the HFS group, in all of the patients, an arterial offender was recorded, while in the GPN group, the only one patient had a mixed offender (*p* < 0.0001). Mixed cases were excluded from this specific analysis, but in the mixed cases (TN and HFS), arterial offenders were also detected.

**Figure 5:**
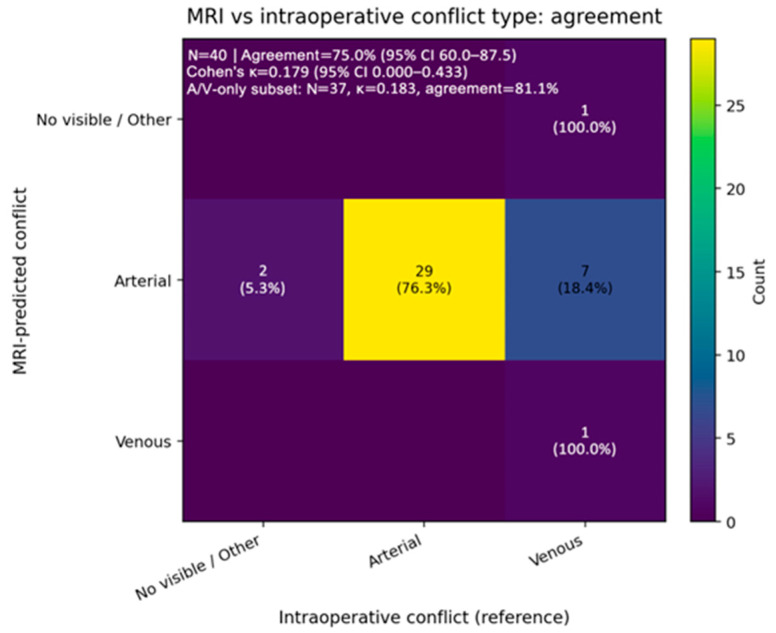
Preoperative MRI evaluation versus intraoperative conflict type in our study (95% CI 60.0–87.5).

A more detailed analysis of the offender in the NVCS from our study group was conducted. One patient could have more than one offender; thus, the sum must not necessarily be 100%. Therefore, we concluded that the superior cerebellar artery (SCA) was the most common offender vessel in our study, followed by the anterior inferior cerebellar artery (AICA). An interesting discrepancy was concluded when comparing the preoperative radiologist-recorded offender and the intraoperative findings, as the MRI-predicted arterial conflicts in seven cases that turned out to be venous conflicts when assessed intraoperatively (Figure 5).

**Figure 6:**
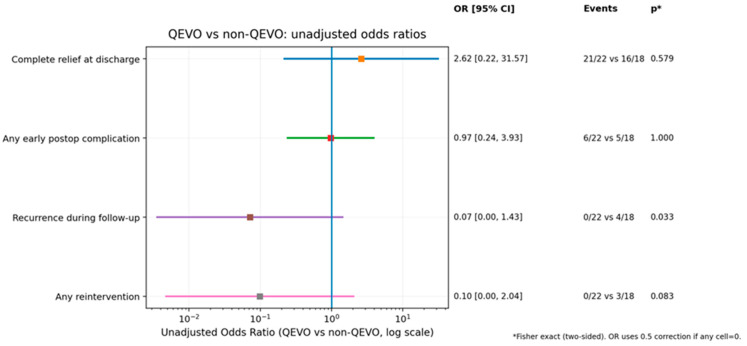
Comparison of outcomes in two groups of patients: the group in which QEVO endoscopy was utilized intraoperatively versus the group of patients treated without adjuvant endoscopy.

One of the most important conclusions from our study regards the utilization of intraoperative endoscopy. In the assessed cohort, patients treated with the aid of adjuvant QEVO had significantly lower recorded recurrences in comparison to the group of patients who were treated without QEVO endoscopy (*p* = 0.033) (Figure 6).

**Figure 7:**
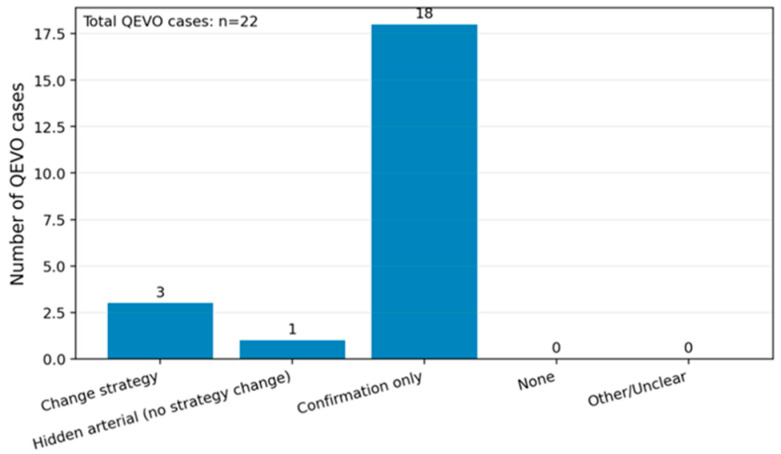
The intraoperative added value of QEVO endoscopy in our cohort.

When analyzing the intraoperative importance of the endoscopy-assisted micro-inspection tool, we found that not only was a hidden arterial conflict revealed, but there was also a change in the surgical strategy, as the endoscope identified additional vascular and/or arachnoid structures that needed supplementary decompression or repositioning (Figure 7).

**Figure 8:**
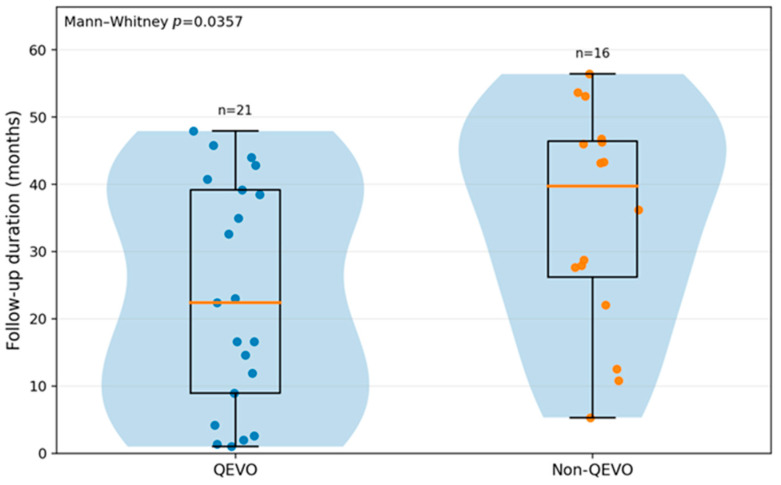
Follow-up duration (months) distributed by QEVO use in the entire cohort (*p* = 0.0357).

The follow-up duration was also different between the QEVO and non-QEVO groups, as shown in Figure 8, and the difference between the two groups was statistically significant (*p* = 0.0357). In the entire cohort, three patients were lost to follow-up: one patient with TN in the QEVO group and two patients with TN in the non-QEVO group.

**Figure 9:**
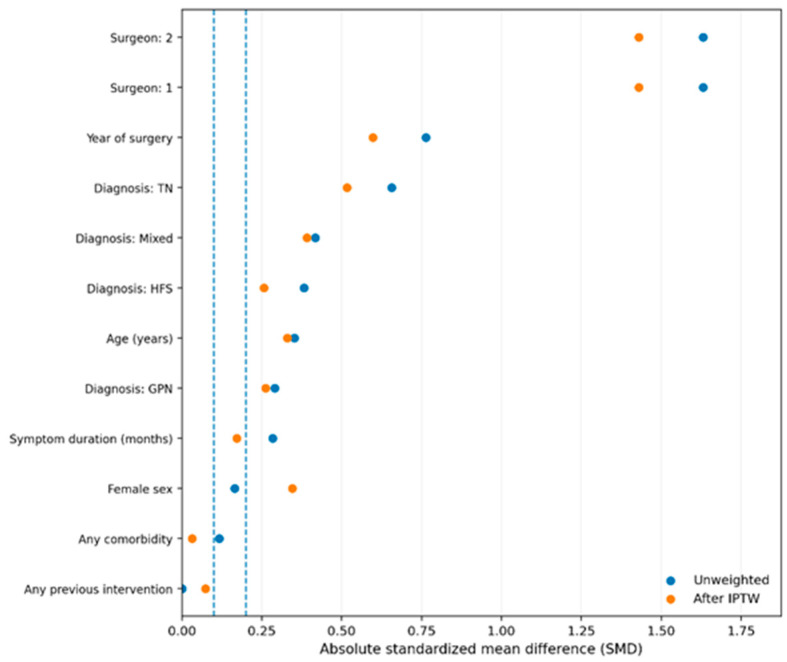
Balance between the QEVO and the non-QEVO groups before and after the propensity score.

Notwithstanding the finding that the QEVO endoscope improved the postoperative outcomes in our study population, its use was not necessarily randomly attributed, but rather was dependent on factors like the surgeon, type of NVCS, or time. Therefore, to account for potential confounding due to non-random allocation, the statistical standard method of inverse probability of treatment weighting (IPTW; propensity score) was used (Figure 9). We acknowledge that surgeon discretion and temporal adoption may still introduce residual confounding, which represents a limitation of the study.

**Figure 10:**
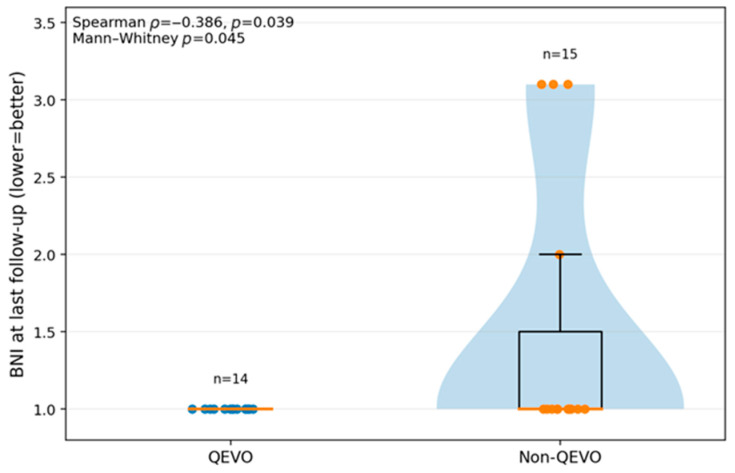
BNI score recorded at the last follow-up in patients with pure TN from our study group. The TN patients were divided into two groups: those treated with endoscope-assisted QEVO and those without.

The Barrow Neurological Institute (BNI) pain intensity score was used in order to assess the symptoms in patients with TN. A total score of I represents the minimum, no pain and no medication, and is the best result that can be obtained, while a score of V is the maximum and shows severe pain or no pain relief after intervention. We analyzed the BNI scores at the last follow-up in patients with pure TN, treated with endoscope-assisted QEVO and without, and the results are shown in Figure 10. The *p*-value was 0.039; therefore, the correlation was statistically significant. One patient with TN from the QEVO group was lost to follow-up.

## 5. Discussions

Despite being a benign condition, many NVCSs greatly affect daily activities, social life, and overall quality of life for the vast majority of patients [75]. The current study comprised a total of 40 patients, of whom 32 patients (80.0%) had TN, 5 patients had HFS (12.5%), 1 patient (2.5%) had GPN, and 2 patients (5.0%) had a mixed NVCS (TN and HFS). Therefore, most of the patients from our cohort had TN.

It has been stated that the diagnosis is based on the clinical criteria established by the International Headache Society, which highlights the presence of three types of TN [76]. While the classical form of TN is characterized by a direct neurovascular conflict, the secondary TN appears due to the presence of an external biological or mechanical factor, such as a tumor or an autoimmune disease like multiple sclerosis. Finally, the third type of TN is the idiopathic one, and in these cases, there were no identified causes [76]. In our study group, all of the included patients had classical TN.

When comparing the mean age, in the TN group it was 59.2 years (+/−11.0), while in the HFS group, the recorded mean age was 47.4 years (+/−9.7), and the difference was statistically significant (*p* = 0.0431). This result suggests that patients with HFS tend to be younger in comparison to patients with TN.

Another interesting statistically significant difference (*p* = 0.0012) was concluded from our study regarding NVCS and smoking status. More than half of patients with TN never smoked (53.1%), and 25% were current smokers, while 40% of patients with HFS were current smokers and 40% were former smokers. However, it is important to mention that the smoking status distribution could also be very different on a wider scale, given the small sample size of patients with HFS.

When analyzing the number of patients with previous interventions prior to presentation in our clinic, we concluded that all of the HFS patients had received previous interventions (botulinum toxin injections in all cases and previous MVD in one case), in comparison to patients with TN, of whom approximately 47% had received previous interventions. This difference was statistically significant (*p* = 0.0498).

Regarding the age distribution of the 40 patients in the analysis, the differences were not statistically significant, but this is more likely due to the small number of patients with HFS and GPN.

Regarding the symptom duration, the median recorded in the TN group was 48 months, and in the HFS group, it was 72 months; therefore, we concluded that HFS tends to have a longer period of symptom presence in comparison to TN. In GPN, the comparison cannot be made, given the singularity of the case. However, the difference was not statistically significant, most likely due to the small number of patients with HFS and GPN (*p* = 0.3702).

Regarding the offender factor discovered in the intraoperative settings, the results were quite similar. In TN, 22 cases had an arterial offender and 9 venous offenders, while in HFS, all of the patients had an arterial offender. Although the patient with GPN had a mixed offender (thick arachnoid adhesions and AICA), the statistical difference between the groups was significant (*p* < 0.0001). It is worth mentioning that the exact conditional value of *p* using chi-square ordering was also significant (0.0208), and the *p*-value for Fisher–Freeman–Halton exact two-sided test was 0.0456. Thus, although the initial *p*-value could be biased given the groups with very small sample sizes, after these tests, the difference is still significant. Nevertheless, the inclusion of the patient with GPN has a major impact on some statistical analyses, which is why Table 5 and some figures include only patients with TN and HFS.

When analyzing the concordances between the preoperative MRI-predicted neurovascular conflict and the intraoperative discoveries, we made three comparable categories. For MRI, it could be venous (2), arterial (1), and not visible (0), and in the intraoperative settings, we categorized them as venous (2), arterial (1), and other (0) for arachnoid-only and mixed (if it is not pure arterial or pure venous). Therefore, in Figure 5, the “not visible/other” appears as a common category. For MRI, this means that there is no detectable conflict, while intraoperatively, it means that the conflict was due to factors other than pure arterial or pure venous ones. The conclusion was that the agreement between the preoperative MRI and the intraoperative settings was 75% (30 patients out of 40). However, despite the agreement percentage being high, the Kappa is low, approximately 0.179, which eliminates the chances of matching the preoperative with the intraoperative just by luck. Thus, it is safe to conclude that even after chance-corrected agreement, the most recorded differences involved the cases predicted by MRI as arterial conflicts but detected intraoperatively as venous conflicts. Intraoperatively, nine patients had a venous offender, but the preoperative MRI only correctly predicted one, which allows us to say that in our study, MRI demonstrated a low sensitivity for venous offenders. A total of seven venous offenders were wrongly classified as arterial on MRI, while one was reported as not visible by the radiologist. It is important to mention that, in our cohort, all HFS cases were arterial, while venous offenders were observed only in TN.

The main focus of the current article points to the added value of the intraoperative adjuvant endoscope—in our case, the QEVO tool. When comparing the outcomes in patients in which the endoscope was used along with the intraoperative microscope, and in patients in which only the microscope was used, we found that the recurrence in the first group was 0 out of 22, while in the second group it was 4 out of 18. The difference was statistically significant, *p* = 0.033, in Fisher’s analysis. The OR was 0.07, and because it is less than 1, it suggests that QEVO endoscopy-assisted MVD is associated with a lower risk of recurrence. Other associations between the two groups, such as complete relief at discharge, any early postoperative complications, or any reinterventions, were not statistically significant and therefore will not be discussed.

When further analyzing the exact added value of the endoscopy in the intraoperative settings in the 22 patients from the group, we found that the endoscope not only identified hidden conflicts that were previously not visible, but also changed the surgical technique. This was due to the fact that, in some patients, it helped identify the need for supplementary decompression, repositioning of the Teflon, or identifying another offender. Furthermore, the endoscope helped confirm the decompression, the correct positioning of the Teflon, and confirmed that there was no residual conflict at the end of the neurosurgical intervention. Therefore, in our study group, the endoscope had a high impact in 4 out of 22 cases (18.2%), and had a confirming role in 18 out of 22 cases (91.8%). It is worth mentioning that there were no reported cases in which the endoscopic assistance did not add any value in our cohort.

Regarding the follow-up duration in our cohort, we analyzed whether there was any difference between the QEVO and non-QEVO groups. In the QEVO group, 21 patients had an available follow-up, while in the non-QEVO group, 16 patients had an available follow-up; so, in total, 37 patients had an available follow-up. After the statistical analysis, we found that in the QEVO group, there was a shorter median follow-up period in comparison to the non-QEVO group (22.4 months versus 39.7 months) (*p* = 0.0357). But why is this result important for our study? It is very important to note that if the QEVO group had a shorter follow-up period, there is a chance that the available time to develop any tardive recurrence is also shorter; thus, the differences between the recurrence rates could also be influenced by the fact that the QEVO group was followed for a shorter amount of time. Nevertheless, this does not mean that this statistical association is false, but we find it important to mention that it could be an existing impacting factor.

Regarding the criteria for QEVO utilization, it is worth mentioning that the endoscopy use was decided in the intraoperative settings based on the surgeon’s judgment when microscopic visualization was limited, when an additional offender was suspected, or when there was a complex neurovascular configuration. Furthermore, in order to assess the baseline compatibility between QEVO and non-QEVO groups, we added a baseline comparison table (Table S1 in Supplementary Materials), which demonstrates that the endoscopy allocation was strongly influenced by the surgeon and year of surgery.

One important aspect to be considered when discussing the use of QEVO-assisted microvascular decompression in our study population is that it was not randomly attributed. The use of QEVO endoscopy was dependent on the surgeon, time, and type of NVCS, as we already stated. These recordings could unbalance the analysis; thus, we considered balancing the groups using IPTW. Before the IPTW, the groups were less comparable, while after the IPTW, the groups were more comparable. On the y-axis, each variable (year of MVD surgery, surgeon, age, etc.) has two points. The x-axis shows the absolute standardized mean difference (SMD), where 0 means perfectly balanced, 0.1 (the pointed line) means low imbalance (acceptable), and 0.2 means a clear imbalance. In this case, our analysis gave three conclusions. The first conclusion is that QEVO endoscopy was not randomly used, as there is an imbalance at baseline, specifically on variables like surgeon, year of intervention, and possibly diagnosis. The essence here is that without adjustment, the comparison between QEVO and non-QEVO is potentially biased. The second conclusion is that IPTW reduces a part of the imbalance, and the third conclusion is that there is a strong clustering on the surgeon variable. This means that the IPTW can improve the balance, but cannot fully repair the lack of overlap. Thus, Figure 9 justifies the method and also offers another limitation for our study, as although improved baseline balance was observed, residual imbalance may persist.

The last analysis of the cohort regards the BNI scores at the last follow-up in patients with pure TN, distributed by the use of the QEVO endoscope. We wanted to see if the BNI score was different between the two groups at the last recorded follow-up. After analyzing, we found that all of the patients in the QEVO group had uniformly favorable BNI scores, while in the non-QEVO group, there were patients with residual pain or incomplete control of pain. The correlation was statistically significant, with a recorded *p*-value of 0.045 (Mann–Whitney test was used because the distribution was not normal). However, given that the N is small and there are many ties to 1, the result could be considered borderline (almost 0.05), and even if it is significant, it should be cautiously reported.

### Study Limitations

Notwithstanding the briefly mentioned limitations, we would like to highlight and detail the shortcomings of our manuscript. Our study has a retrospective design; thus, it cannot demonstrate causality on its own, and the utilization of QEVO endoscopy was not randomized, but was decided in the intraoperative settings, which induces a selection bias. Furthermore, the endoscope was used predominantly by one neurosurgeon, especially in the most recent years, as the technique was progressively embraced. This can impact the outcomes through factors that are endoscopy-dependent, such as technique, expertise, learning curve, etc. Although we tried to attenuate it through baseline comparison, SMD, and IPTW, it can remain a residual confounder.

Another important previously mentioned limitation is represented by the small cohort size and the rarity of the studied events. The study group comprises 40 consecutive patients, and even smaller subgroups. The complications and late events are rare; thus, the CIs are large, and the statistical significance is limited. Certain statistically significant results might be unstable, and some real differences may remain undetected.

The follow-up for time-dependent outcomes is uneven or incomplete, and it is not identical between the groups, which could influence the recurrences and reinterventions. However, we specifically reported the N follow-up (we reported how many patients had an available follow-up), and the median follow-up was 28.8 months. In addition, we included analyses for sensitivity and cautious interpretation.

Regarding the generalizability, a single-center study may reflect the work of the team and the operative flux; therefore, the results need external validation, ideally in prospective and multicentric studies, before being considered generalizable. For GPN/mixed cases, we could not draw robust statistical conclusions, but we can mainly offer descriptive results. Some variables are based on clinical documentation and can have variability (such as smoking—not reported).

## 6. Illustrative Case Showcasing the Added Value of the Intraoperative Endoscopic Micro-Inspection Tool in Recurrent Trigeminal Neuralgia

Similarly to the current neurosurgical literature, our personal experience of the use of MVD for NVCS clearly supports the intraoperative importance of the endoscopic micro-inspection tool. A relevant case from personal archives is that of a 45-year-old female patient who had previously undergone classical MVD for TN one year before presentation. The patient was surgically treated in another Neurosurgical Center and was admitted to our clinic with recurrent pain in the left V2 and V3 distribution areas, reporting that her symptoms returned three months after the initial surgery, and were treated with Carbamazepine.

**Figure 11:**
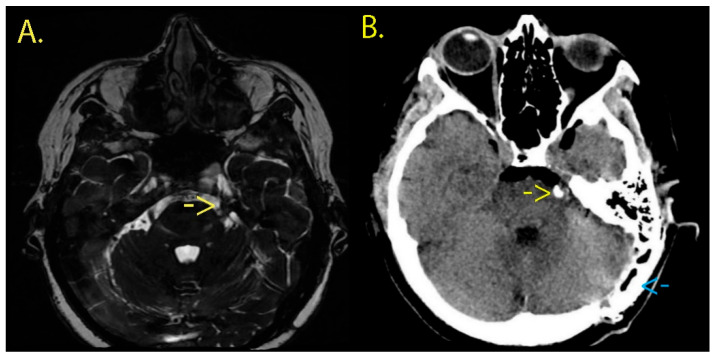
(**A**) Preoperative FIESTA-C sequence brain MRI revealing a neurovascular conflict on the left side (yellow arrow) between the left trigeminal nerve and left SCA. (**B**) Postoperative cerebral CT scan on the first day. The yellow arrow points out the new Teflon piece, while the blue arrow shows the cranioplasty cement used to cover the postoperative skull defect.

The preoperative MRI examination revealed a residual neurovascular conflict involving the superior cerebellar artery (SCA) on the left side (Figure 11), and after discussing the therapeutic options available, she consented to neurosurgical reintervention. It is worth mentioning that this case is presented as an illustrative workflow example of the use of an endoscopic micro-inspection tool and intraoperative verification, rather than as standalone evidence of superiority.

**Figure 12:**
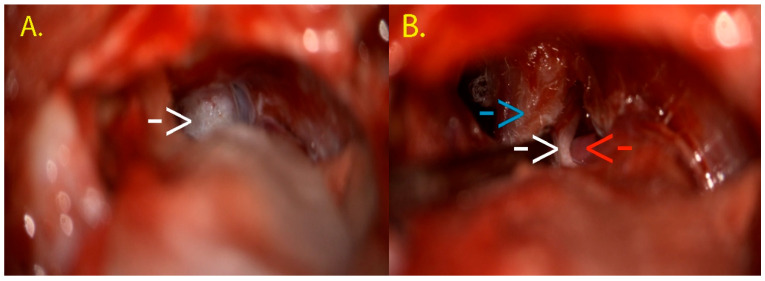
(**A**) Initial aspect of the inspection of the left cerebellopontine angle; the white arrow shows the Teflon piece from the first neurosurgical intervention. (**B**) Dissection of the old Teflon piece with uncovering of the trigeminal nerve (white arrow). The old Teflon piece can be seen on the left and above the nerve (blue arrow), while the SCA is under the nerve (red arrow). This figure illustrates the revision workflow and endoscopic-assisted verification; conclusions regarding endoscopic added value are derived from the cohort-level analyses.

Intraoperatively, the QEVO endoscopic micro-inspection tool was used as an adjunct to the operating microscope. The main goal of the initial dissection was represented by the cautious exploration of the Teflon piece inserted during the previous neurosurgical intervention. The old Teflon piece covered the trigeminal nerve, with the SCA loop resting on the posterior aspect of the nerve, at the REZ. The Teflon was then safely dissected (Figure 12).

**Figure 13:**
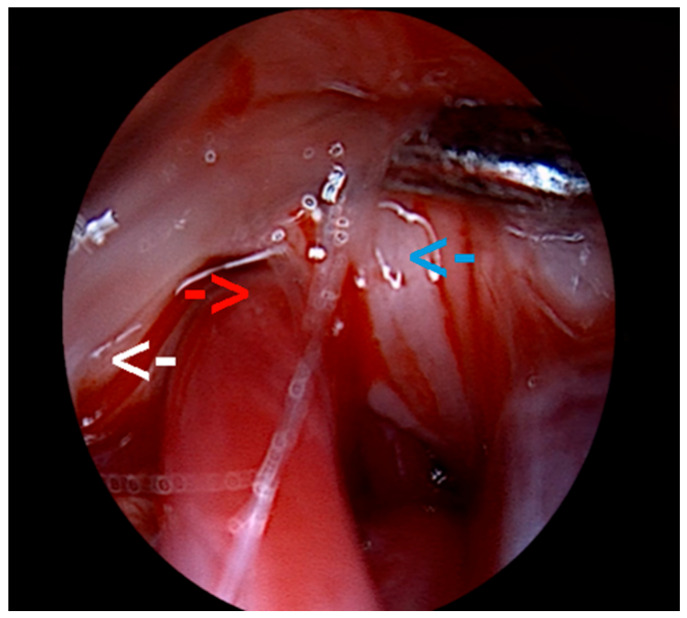
Intraoperative visualization with QEVO micro-inspection tool. The white arrow points out the brainstem, while underneath the double-ended blunt tissue elevator, the REZ of the trigeminal nerve can be seen (blue arrow). Finally, the red arrow shows the SCA mechanically conflicting with the trigeminal nerve at its entry zone from the brainstem.

The reason behind this careful exploration was the frequent reports of Teflon-formed adhesions or granulomas as one of the most common causes of treatment failure [30]. However, after a thorough inspection, we excluded a Teflon-related conflict, and we reinspected the anatomical area, this time using the QEVO endoscope, only to identify a previously undetected neurovascular conflict between the SCA and the posterior aspect of the left trigeminal nerve at the root entry zone (REZ) (Figure 13). These findings concluded that the recurrence of symptoms in our patient was due to an incomplete initial decompression.

**Figure 14:**
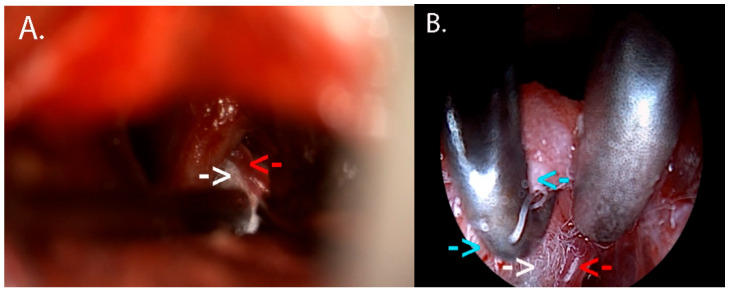
(**A**) This image highlights the mobilization of the SCA loop (red arrow) and the positioning of the Teflon piece (white arrow) between this artery and the trigeminal nerve. (**B**) Final inspection using the QEVO micro-inspection endoscope in order to assess the optimal placement of the Teflon piece and the nerve decompression. Underneath the surgical hook, there is the trigeminal nerve (blue arrow), under the surgical suction cannula, there is SCA (red arrow), and in between them, there is the Teflon piece (white arrow).

Afterwards, a new piece of Teflon was placed in order to solve the newly discovered conflict, and the trigeminal nerve was finally decompressed. At the end of the surgical intervention, the QEVO endoscope was reinserted to confirm the correct placement of the Teflon piece (Figure 14).

It is important to mention that there were no complications related to the use of the endoscope, and the tool was used for a total time of less than a minute, without disrupting the neurosurgical flow or prolonging the operative time. On the ninth day after this intervention, the patient developed a cerebrospinal fluid (CSF) fistula at the site of the surgical wound, which is a very commonly described complication after MVD procedures in NVCS. Nonetheless, this related complication was successfully managed with a lumbar drain, without impacting the overall outcome, and almost two years after the intervention, the patient remained completely pain-free.

The presented case illustrates how endoscopic micro-inspection can be incorporated as an adjunct for intraoperative verification, particularly in revision settings. Nevertheless, the study’s conclusions regarding the added value are based on aggregate cohort findings rather than a single illustrative observation.

## 7. Conclusions

QEVO-assisted endoscopy can add value in MVD surgery for patients diagnosed with NVCS, in selected cases, based on the neurosurgeon’s judgment. Its utility is highlighted especially when it comes to visualization, as it can identify conflicts that were previously unseen with the microscope alone, can confirm the positioning of Teflon, and can identify additional neurovascular conflicts, impacting the neurosurgical technique. Although the main limitation of our study regards the small number of patients with HFS and GPN, many of the results support the value of endoscopy-assisted tools.

## Figures and Tables

**Table 1:** Epidemiology of the most common NVCSs currently reported in the medical literature as reviews or clinical series. ASR = Age-Standardized Rate (or Age-Adjusted Rate); AJNR = American Journal of Neuroradiology. * These reported numbers are a reflection of the selection criteria of a department or institution, and they do not necessarily represent the numbers from the general population.

Type of NVCS	Reported Incidences	Reported Prevalences	Reporting Study	Studies Reporting only Clinical Series *
Trigeminal neuralgia	25.33/100.000 (0.0253%/year); approximately 4.3–28.9/100.000/year (0.0043–0.0289%/year)	45.38/100.000 (0.0453%); lifetime prevalence of 108.43/100.000 (0.1084%); 76.8/100.000 (0.0768%)	[10,11]	46.4% (115 cases) in a cohort of 248 patients [12]; 32.4% (34 cases) in a cohort of 105 patients [13]
Hemifacial spasm	0.74/100.000 in men and 0.81/100.000 in women (0.00074–0.00081%/year); (ASR) 1.53/100.000/year (0.00153%/year); (AJNR) approximately 1/100.000/year (approximately 0.001%/year)	7.4/100.000 in men and 14.5/100.000 in women (0.0074–0.0145%); (ASR) 10.62/100.000 (0.01062%)	[1,11,14]	49.6% (123 cases) in a cohort of 248 patients [12]; 56.2% (59 cases) in a cohort of 105 patients [13]
Glossopharyngeal neuralgia	0.2–0.4/100.000/year; 0.0002–0.0007%/year in some reports; (AJNR) 0.2–0.7/100.000/year	Approximately 0.2–1.3% of all NVCS	[1,15]	4.0% (10 cases) in a cohort of 248 cases [12]; 11.4% (12 cases) in a cohort of 105 patients [13]
Vestibular paroxysmia	(AJNR) Unknown incidence	Affects less than 1 in 2000 people; approximately 3% of patients diagnosed with vertigo	[1,16,17]	-

**Table 2:** Summary of reported outcomes of MVD in patients with GPN.

Study (Year)	Sample Size	Immediate Postoperative Relief	Long-Term Relief	Follow-Up
Xia et al. (2018) [40]	228 patients	89.5%	86.9% at more than 5 years	54.3 months
Sampson et al. (2004) [41]	47 patients	98%	28 out of 29 patients were pain-free (only 29 patients were available for long-term follow-up)	Median 12.7 years
Zhao et al. (2017) [42]	35 patients	94.3%	28 out of 30 patients were pain-free (only 30 patients were available for long-term follow-up)	Variable
Ferroli et al. (2009) [43]	31 patients	-	90.3%	Mean 7.5 years
Revuelta-Gutiérrez et al. (2016) [44]	14 patients	-	92.2%	26 months

**Table 3:** A summary of the most relevant studies regarding the efficacy of endoscopy in MVD surgery for NVCS.

Study (Year)	Study Description	Number of Patients and Type of NVCS	Main Results
Rak et al., Endoscope-assisted microsurgery for microvascular compression syndromes (2004) [55]	Series—endoscope-assisted MVD	28 patients: 17 TN, 10 HFS, and 1 VP	The endoscope has been proven very useful in cases of venous compression (such as trigeminal vein in Meckel’s cave), multiple compression points, and in verifying the decompression and the Teflon positioning
Lee et al., Endoscopic vs microscopic MVD for TN (2017) [56]	A single-surgeon comparative retrospective study	167 patients: 93 with microscopic MVD, and 74 with endoscopic MVD	The outcomes regarding pain relief were similar, but fewer patients had postoperative headaches after one month
Shu et al., Endoscopic Versus Microscopic Microvascular Decompression for Trigeminal Neuralgia: A Prospective Controlled Study (2024) [57]	Controlled prospective study	52 patients: 23 with assisted endoscopy, and 29 with microscopic MVD	Similar pain results at 12 months follow-up; more offending vessels identified in patients were the endoscope was used; no reported mortality
Chen et al., Endoscopic microvascular decompression versus microscopic microvascular decompression for trigeminal neuralgia: A systematic review and meta-analysis (2023) [50]	Systematic review and meta-analysis	Includes 3 studies, 442 patients: 218 treated with endoscopic MVD, and 224 with microscopic MVD	Pain relief and complications without statistically significant differences between the groups in their study
Zagzoog et al., Endoscopic versus open microvascular decompression for trigeminal neuralgia: a systematic review and comparative meta-analysis (2018) [58]	Systematic review and meta-analysis	Includes 23 articles, 6749 patients, reporting pain relief, recurrences, and complications	Similar pain relief results and fewer recurrences in patients treated with adjuvant endoscopy (however, the differences were not statistically significant); complication rates reported major differences: 19% in classic MVD versus 8% in endoscopic MVD (statistically significant results)
Bohman et al., Fully endoscopic microvascular decompression for trigeminal neuralgia: technique review and early outcomes (2014) [59]	Prospective study, fully endoscopic series	47 patients with TN	Excellent pain relief in 94% of the cases (from 10 preoperatively to 0 postoperatively), *p* < 0.0001; pain interference had dramatically decreased with reduction in Brief Pain Inventory Scores (*p* < 0.0001); low morbidity rate—2% (only one case of permanent hearing loss)
Karadag et al., Endoscope Assisted Microvascular Decompression for Trigeminal Neuralgia Surgical Safety and Efficacy (2024) [60]	Cadaver dissection and illustrative case	5 cadavers (10 parts) and 1 illustrative case of TN	Supports the conclusions of better visualization and less cerebellar retraction in the corridor of the cerebellopontine angle
Jarrahy et al., Endoscope-assisted microvascular decompression of the trigeminal nerve (2000) [61]	Retrospective case series	21 patients with TN	14 conflicts were identified only after endoscopic inspection, while in 5 patients in which the endoscopy was used, additional surgical maneuvers were required in order to completely decompress the nerve, as it was insufficiently decompressed initially
Teo et al., Endoscope-assisted microvascular decompression for trigeminal neuralgia: technical case report (2006) [62]	Large retrospective series, endoscope-assisted MVD	114 patients with TN	Endoscopy improved visualization of hidden arterial and venous conflicts; complete pain relief was reported in 99.1% of patients at a mean follow-up of approximately 29 months; complication rates were comparable to microscopic MVD
Tang et al., Endoscope-assisted neurovascular decompression of the trigeminal nerve: a cadaveric study (2013) [63]	Cadaveric anatomical and technical study	7 cadaveric heads (TN model)	Endoscope-assisted MVD provided superior maneuverability and visualization of the cerebellopontine angle, particularly in regions inaccessible to the microscope
Setty et al., Endoscopic vascular decompression for trigeminal neuralgia: clinical outcomes and technical note (2014) [64]	Prospective study (fully endoscopic)	57 patients with TN	Immediate postoperative pain control (BNI I–III) was achieved in all patients; durable pain relief was maintained in approximately 98% of patients at follow-up; low morbidity rate
Dubey et al., Full Endoscopic Vascular Decompression in Trigeminal Neuralgia: Experience of 230 Patients (2018) [65]	Retrospective study (large series), fully endoscopic	230 patients with TN	Complete pain relief was achieved in 88.7% of patients; satisfactory improvement in 5.8%; recurrence observed in 6.5% during long-term follow-up; complications were generally transient
Xiang et al., Prospective Study of Neuroendoscopy versus Microscopy: 213 Cases of Microvascular Decompression for Trigeminal Neuralgia Performed by One Neurosurgeon (2018) [66]	Prospective controlled study	213 patients with TN	No statistically significant differences between endoscopic and microscopic groups regarding pain relief at 1 year or major postoperative complications
Li et al., A Meta-Analysis of Endoscopic Microvascular Decompression versus Microscopic Microvascular Decompression for the Treatment for Cranial Nerve Syndrome Caused by Vascular Compression (2019) [67]	Systematic review and meta-analysis	9 studies, 1093 patients with TN, HFS, and GPN	Endoscopic MVD showed higher rates of offending vessel identification and lower perioperative complication rates, with comparable or improved long-term symptom relief
Luzzi et al., Endoscope-Assisted Microneurosurgery for Neurovascular Compression Syndromes: Basic Principles, Methodology, and Technical Notes (2019) [68]	Case series and technical classification	43 patients: 25 TN, 9 HFS, 2 GPN, and other NVCS	Endoscopy was essential or highly useful in complex conflicts; complete symptom resolution was reported; improved visualization in deep neurovascular corridors
Wang et al., Complete neuroendoscopic versus microscopical trigeminal neuralgia microvascular decompression (MVD) in primary trigeminal neuralgia (PTN) (2021) [69]	Prospective comparative study	87 patients (45 endoscopic, 42 microscopic)	Endoscopic MVD demonstrated higher efficacy, lower complication rates, and lower recurrence rates at 1-year follow-up
Wurzinger et al., Long-term outcome of endoscope-assisted microvascular decompression in trigeminal neuralgia (2025) [51]	Retrospective study	182 patients with classical TN	Endoscope-assisted MVD was safe and effective, with high long-term pain-free rates; endoscopy was particularly useful when microscopic visualization of Meckel’s cave was limited
Feng et al., Endoscopic microvascular decompression for primary trigeminal neuralgia: surgical experience and early outcomes (2025) [70]	Retrospective cohort	137 patients with TN	Effectiveness rates were higher in the endoscopy group
Pham et al., Intraoperative endoscope view classification of neurovascular compression in hemifacial spasm condition: a single neurosurgeon prospective cross-sectional study (2023) [71]	Prospective cross-sectional study	29 patients with HFS	The intraoperative analysis showed that in 41.3% of the cases, the neurovascular conflict was missed without the endoscope
Zheng et al., Fully endoscopic microvascular decompression for hemifacial spasm: a clinical study and analysis (2024) [72]	Retrospective study (fully endoscopic)	16 patients with HFS	Immediate symptom relief in 93.75% of the patients, with no recurrences and no mortality at the 2-month follow-up
Jiang et al., Fully endoscopic microvascular decompression for hemifacial spasm (2022) [73]	Retrospective study (fully endoscopic)	5 patients with HFS	Symptoms amelioration in all cases (reported efficacy 100%)
Liu et al., Efficacy and safety of microvascular decompression for hemifacial spasm: Endoscopy vs microscopy (2026) [74]	Retrospective study	189 patients with HFS	Similar efficacy at 1 year (*p* = 0.709), without significant differences recorded in complications; the endoscopic approach had a more rapid learning curve, with less postoperative vertigo reported (*p* = 0.037)

**Table 4:** A descriptive table presenting the demographic profile and clinicopathological characteristics in our study group.

Characteristic	Overall Cohort (*N* = 40)
**Age, years (mean +/− SD)**	57.3 +/−11.4
**Age, years (median)**	56.5 [(49–67)
**Female sex, *N* (%)**	24 (60.0%)
**Type of NVCS, *N* (%)**	
**TN**	32 (80.0%)
**HFS**	5 (12.5%)
**GPN**	1 (2.5%)
**Mixed (TN and HFS)**	2 (5.0%)
**Side, *N* (%)**	
**Left**	21 (52.5%)
**Right**	19 (47.5%)
**Symptom duration, months (median)**	48 (12–87)
**Symptom duration, months (range)**	2–180
**MRI neurovascular conflict, *N* (%)**	
**Arterial**	38 (95.0%)
**Venous**	1 (2.5%)
**No visible conflict on MRI**	1 (2.5%)
**Intraoperative neurovascular conflict, *N* (%)**	
**Arterial**	29 (72.5%)
**Venous**	9 (22.5%)
**Arachnoid adhesions only**	1 (2.5%)
**Mixed: arachnoid adhesions and vascular (arterial)**	1 (2.5%)
**QEVO micro-inspection endoscope used, *N* (%)**	22 (55.0%)
**QEVO usage by diagnosis, *N* (%)**	
**TN**	15 (68.2%)
**HFS**	4 (18.2%)
**GPN**	1 (4.5%)
**Mixed (TN and HFS)**	2 (9.1%)
**Timing of QEVO usage, *N* (%)**	
**Exploration and final inspection**	16 (72.7%)
**Final inspection only**	5 (22.7%)
**Exploration only**	1 (4.5%)
**MVD material, *N* (%)**	
**Teflon**	37 (92.5%)
**Alternative padding**	1 (2.5%)
**Other (e.g., arachnolysis/coagulation)**	2 (5.0%)
**Previous interventions, *N* (%)**	20 (50.0%)
**Type of previous intervention, *N* (%)**	
**Prior MVD**	3 (7.5%)
**Prior rhizotomy or destructive procedure**	9 (22.5%)
**Local infiltrations**	3 (7.5%)
**Prior Gamma Knife**	2 (5.0%)
**Prior botulinum toxin injection**	5 (12.5%)
**Other interventions (acupuncture, oxygen therapy, etc.)**	3 (7.5%)
**Smoking status, *N* (%)**	
**Never**	19 (47.5%)
**Current**	10 (25.0%)
**Not reported**	9 (22.5%)
**Former**	2 (5.0%)
**Early postoperative outcome at discharge, *N* (%)**	
**Complete relief**	37 (92.5%)
**No relief**	2 (5.0%)
**Partial relief**	1 (2.5%)
**Any early postoperative complication, *N* (%)**	11 (27.5%)
**Persistent complications at the last follow-up (trigeminal hypoesthesia)**	1 (2.5%)
**Transient complications**	10 (25.0%)
**Selected complications, *N* (%)**	
**CSF leak**	4 (10.0%)
**Meningitis**	1 (2.5%)
**Suture granuloma**	2 (5.0%)
**Facial palsy**	1 (2.5%)
**Abducens palsy**	1 (2.5%)
**Diplopia**	1 (2.5%)
**Cerebellar ischemia**	1 (2.5%)
**Trigeminal hypoesthesia**	1 (2.5%)
**Follow-up duration, months (median) (*N* = 37)**	28.8 (12.5–43.3)
**Follow-up duration, months (range) (*N* = 37)**	1.0–56.4
**Patients lost to follow-up, *N* (%)**	3 (7.5%)
**Recurrence during follow-up, *N* (%)**	4 (10.0%)
**Reintervention (MVD surgery), *N* (%)**	2 (5.0%)
**Reintervention (other: Gamma-Knife Radiosurgery)**	1 (2.5%)

**Table 5:** Statistical differences and similarities between patients with TN and HFS.

Characteristic	TN (*N* = 32)	HFS (*N* = 5)	*p*-Value
**Age, years (mean ± SD)**	**59.2** ± **1.0**	**47.4 ± 9.7**	**0.0431**
**Female sex, *N* (%)**	19 (59.4%)	3 (60.0%)	1.0000
**Left side, *N* (%)**	18 (56.2%)	2 (40.0%)	0.6443
**Symptom duration, months (median)**	48.0 [12.0–69.0]	72.0 [36.0–84.0]	0.5464
**MRI conflict on MRI, *N* (%)**			0.8477
**Arterial**	30 (93.8%)	5 (100.0%)	
**Venous**	1 (3.1%)	0 (0.0%)	
**No visible conflict**	1 (3.1%)	0 (0.0%)	
**Intraoperative conflict, *N* (%)**			0.3428
**Arterial**	22 (68.8%)	5 (100.0%)	
**Venous**	9 (28.1%)	0 (0.0%)	
**Arachnoid adhesions only**	1 (3.1%)	0 (0.0%)	
**QEVO used, *N* (%)**	15 (46.9%)	4 (80.0%)	0.3398
**Any previous intervention, *N* (%)**	**15 (46.9%)**	**5 (100.0%)**	**0.0498**
**Smoking status, *N* (%)**			**0.0012**
**Never**	17 (53.1%)	0 (0.0%)	
**Current**	8 (25.0%)	2 (40.0%)	
**Former**	0 (0.0%)	2 (40.0%)	
**Not reported**	7 (21.9%)	1 (20.0%)	
**Any comorbidity, *N* (%)**	23 (71.9%)	5 (100.0%)	0.3067

## Data Availability

The data presented in this study are available on request from the corresponding author.

## Supplementary Materials

The following supporting information can be downloaded at https://www.mdpi.com/article/10.3390/neurolint18040066/s1: Table S1. Baseline comparison table for QEVO versus non-QEVO cases. SMD—standardized mean difference.
