# Supplementary material for: The Added Value of Endoscopic Micro-Inspection in Microvascular Decompression for Trigeminal Neuralgia and Hemifacial Spasm: Literature Review and Single-Center Experience
Source: Neurol Int. 2026 Mar 31;18(4):66. doi: 10.3390/neurolint18040066 (PMC13119251; doi:10.3390/neurolint18040066)
Supplement: Supplementary file 1 [file neurolint-18-00066-s001.zip › neurolint-4186421-supplementary.pdf]

Supplementary Materials

Table S1. Baseline comparison table for QEVO versus non-QEVO cases; SMD - Standardized Mean Difference.

**Characteristic**	**QEVO (n=22)**	**Non-QEVO (n=18)**	**p-value**	**SMD***
Age, years (mean +/-SD)	55.5 +/- 11.2	59.5 +/- 11.5	0.301	0.35
Age, years (median [IQR])	54.0 [49.2-64.2]	62.5 [50.8-67.8]		
Symptom duration, months (median [IQR])	42.0 [19.5-78.0]	54.0 [12.0-114.0]	0.512	0.28
Female sex, N (%)	14 (63.6%)	10 (55.6%)	0.748	0.16
Left side, N (%)	11 (50.0%)	10 (55.6%)	0.761	0.11
Diagnosis (TN/HFS/GPN/Mixed), N (%)			χ^2 p=0.206	0.66
TN	15 (68.2%)	17 (94.4%)		
HFS	4 (18.2%)	1 (5.6%)		
GPN	1 (4.5%)	0 (0.0%)		
Mixed	2 (9.1%)	0 (0.0%)		
Smoking reported, N (%)	16 (72.7%)	15 (83.3%)	0.476	0.25
Ever-smoker (current/former), N (%)	7 (31.8%)	5 (27.8%)	1.000	0.09
Any comorbidity, N (%)	17 (77.3%)	13 (72.2%)	0.731	0.12
Any previous intervention, N (%)	11 (50.0%)	9 (50.0%)	1.000	0.00
MRI conflict type, N (%)			χ^2 p=0.360	0.36
Arterial	21 (95.5%)	17 (94.4%)		
Venous	0 (0.0%)	1 (5.6%)		
None	1 (4.5%)	0 (0.0%)		
Intraoperative conflict type, N (%)			χ^2 p=0.228	0.47
Arterial	18 (81.8%)	11 (61.1%)		
Venous	3 (13.6%)	6 (33.3%)		
Arachnoid	0 (0.0%)	1 (5.6%)		
Mixed	1 (4.5%)	0 (0.0%)		
Year of surgery (median [IQR])	2024.0 [2022.0-2024.8]	2022.5 [2021.2-2023.0]	0.033	0.76
